# Beyond Basic Feedback in Mobile Brief Interventions: Designing SMS Message Content for Delivery to Young Adults During Risky Drinking Events

**DOI:** 10.2196/mhealth.6497

**Published:** 2017-06-20

**Authors:** Cassandra J C Wright, Paul M Dietze, Megan S C Lim

**Affiliations:** ^1^ MacFarlane Burnet Institute for Medical and Public Health Research Centre for Population Health Melbourne Australia; ^2^ Department of Epidemiology and Preventive Medicine School of Public Health and Preventive Medicine Monash University Melbourne Australia; ^3^ Melbourne School of Population and Global Health University of Melbourne Melbourne Australia

**Keywords:** alcohol drinking, young adult, mHealth, text messaging, motivational interviewing, community-based participatory research

## Abstract

**Background:**

Brief interventions can reduce alcohol consumption in young people through screening and delivery of personally relevant feedback. Recently, Web and mobile platforms have been harnessed to increase the reach of brief interventions. Existing literature on mobile-based alcohol brief interventions indicates mixed use of theory in developing interventions. There is no research available to guide the development of SMS text messaging (short message service, SMS) interventions delivered during risky drinking events.

**Objective:**

The aim of this study was to develop and pilot an alcohol-related risk-reduction brief intervention delivered by SMS to Australian young adults during drinking events. This paper describes the development of intervention message content, with specific focus on the context of delivery during drinking events.

**Methods:**

A sample of 42 young adults attended 4 workshops; these comprised focus-group style discussion on drinking habits and motivations, discussion of intervention design, analysis of existing alcohol media campaigns, and participant development of message content. Data were analyzed thematically.

**Results:**

Participants described a focus on having fun and blocking out any incongruent negative influences during drinking episodes. For content to be acceptable, nonjudgmental and non-authoritative language was deemed essential. A preference for short, actionable messages was observed, including suggestions for reminders around drinking water, organizing transport home, checking on friends, and plans the next day. Participants were excited about the potential for messages to be tailored to individuals, as previous alcohol-related campaigns were deemed too generic and often irrelevant. Normative-based messages were also perceived as largely irrelevant as participants felt that they understood the drinking-related norms of their immediate peers already.

**Conclusions:**

Findings from this study offer insights into young adults’ drinking events and practical advice for designing alcohol-related brief interventions. During our formative development process, we demonstrated a neat correspondence between young people’s preferences for alcohol harm reduction interventions and the theoretical principles of brief interventions, including acceptable topics and message style.

## Introduction

Alcohol consumption is a significant public health concern in Australia, particularly in relation to risky single-occasion drinking in young people [1]. Alcohol is consistently reported as a leading cause of disease and injury burden for 15-25-year olds [1,2]. Related harms include physical and sexual assault, suicide, risky sexual behavior, memory loss, blackouts, brain impairment, and cognitive deficits [1,3-5]. In the 2013 Australian National Drug Strategy Household Survey, more than 65% of 18-19-year-olds and 60% of 20-29-year-olds reported engaging in risky single-occasion drinking (defined as 4 or more Australian standard drinks in a single session, or 40 grams of alcohol) [2]. One-third of 18-24-year-olds reported drinking 11 or more standard drinks in a single episode in the past year [2]. In addition, a 2010 study found that 42% of 16-24-year olds from the state of Victoria had consumed more than 20 drinks in one session in the past year [6].

One of the few individual-level prevention strategies known to effectively reduce alcohol consumption in young people is called brief intervention. Well-designed brief interventions are a low-cost strategy to reduce alcohol consumption and harm [7-10]. Brief interventions generally involve screening and a short counseling session, which may include feedback based on screening, education on risk profile, advice for reducing consumption, and discussions around change. The FRAMES (Feedback, Responsibility, Advice, Menu Options, Empathy, and Self-Efficacy) model [7,9,11] provides more detail on evidence-based core components of brief interventions and suggests: provision of personally relevant “feedback,” discussions relating to “responsibility,” nonjudgmental “advice” for changing behavior, suggesting a “menu” of options to support change, expression of “empathy,” and encouraging “self-efficacy.”

Motivational interviewing is a key component of brief interventions [7]. Motivational interviewing is a counseling technique commonly used for health behavior change that involves a nonjudgmental and client-centered approach [11]. A motivational interviewing therapist asks a client the questions designed to elicit their own motivations for changing their behavior and engages them in personalized “change-talk” about their behavior [11]. A 5-min brief intervention that uses these principles has been found to be as effective in reducing alcohol consumption as a 20-min counseling session [12]. A key appeal of brief interventions is its effectiveness among individuals at different stages of readiness to change [7], making it applicable for use within young adults, including university students who are less likely than older adults to be specifically motivated to reduce drinking [13]. Although brief intervention approaches have been successful in changing behaviors, including alcohol consumption and other substance use [7-10,14-18], their scalability has been limited by time and the resources required to implement individualized, face-to-face (albeit brief) sessions [19].

Recently, Web and mobile phone platforms have been harnessed to increase the reach of interventions intended to change behaviors in the realms of sexual health, physical activity, chronic disease management, and alcohol consumption [20-37]. These interventions commonly involve providing health-related messages either through SMS text messaging (short message service, SMS) or mobile phone apps, allowing low-cost yet high-reach delivery of messages to target groups, with some evidence of successful behavior change [20-29,36]. However, as researchers learn to harness these technologies and translate brief interventions to mobile platforms, there has been variable emphasis placed on the theoretical basis for the intervention content [37-41]. Among studies which do publish details on the theoretical basis of interventions, there is diversity in the theoretical frameworks and behavior change techniques which have been applied [38,41,42]; as noted by Abraham and Michie, there is no standardized vocabulary for describing behavior change intervention techniques [43]. Studies of SMS interventions increasingly use the labeling of brief intervention; however, many show limited links between message content and brief intervention or motivational interviewing theory. Brief interventions that have been implemented here are commonly described in the literature as methods using basic screening and feedback components, but the other elements of FRAMES are either not described or not integrated. Though this makes for a more simple intervention, it may perpetuate the understanding that brief interventions are a homogeneous entity [44,45] and deemphasizes the importance of the rest of the brief intervention “toolbox.” A recent study compared several types of brief interventions delivered to college students, including an assessment-only arm, a motivational interviewing-only arm, a feedback-only arm, and a combined motivational interviewing and feedback arm; the study found that the combined motivational interviewing and feedback intervention significantly reduced drinking, whereas none of the other interventions varied from the assessment-only arm [44]. This highlights the potential impact that different message styles might in fact have. The frequent use of feedback-only brief interventions in mobile health (mHealth) may have further implications for developing and interpreting the evidence base around effectiveness of mobile brief interventions for changing behavior such as alcohol consumption. A recent systematic review called for greater transparency in use of theory in developing mobile-based health interventions [38].

The feedback provided within mobile brief interventions has also varied considerably in terms of both content and tone. Many publications describe content developed solely by researchers or clinicians [30,42,46], with limited evidence of consultation with the target group [37,41]. Kristan and Suffoletto found that young people perceived most expert-authored messages to be only somewhat interesting or helpful [47]. Bock et al found that differences in language and construction of alcohol SMS content developed by either the researchers or students affected their interpretation and acceptability [41]. These authors, and others, have strongly advocated for participatory methods to be used in developing mHealth interventions as we continue to disentangle what components of our interventions are acceptable and effective [37,38,41]. Participatory methods are also important for the development of tailored interventions, which require understanding of the target population’s culture, needs, and preferences [37,47-49]. Tailoring is thought to potentially enhance the ability of an intervention to act through personal motivation, which theory and evidence suggest is an important feature of effective brief interventions. However, evidence is thus far limited as to how best to apply complex personalized tailoring in SMS format and further research is hence required [37].

Although other studies have examined young people’s opinions of alcohol interventions [37,47,50,51], this has not previously been studied in the context of in-event intervention delivery, which could potentially be different due to mood, intoxication, attention-span, and social factors.

We developed and piloted an alcohol-related risk-reduction intervention delivered by SMS to Australian young adults during drinking events [52]. This paper describes the development of the message content for this intervention.

## Methods

### Sample and Recruitment

We recruited young adults to participate in the development, testing, and evaluation of the intervention [52]. Participants were recruited through advertising on social media and with hardcopy flyers pinned to bulletin boards at two major universities in Melbourne, Australia. Advertisements called for young people meeting the following eligibility criteria to participate in developing a mobile phone–based intervention aiming to help reduce risky drinking for young people. Stated inclusion criteria included those who (1) were aged 18-25 years, (2) owned a mobile phone and were willing to use it to test intervention, and (3) consumed alcohol at least weekly.

### Procedure

Four development workshops were held in June 2014, with 8-12 participants in each group; each workshop lasted for 3 h. Participants were given an Aus $40 gift card and offered light refreshments. Two groups were mixed gender, one was female only, and one was male only. Participants were allocated to groups depending on preference and availability.

At the beginning of each session, we told participants that we wanted to create a mobile phone–based intervention to be delivered during nights on which they were planning to drink alcohol. We indicated that our plan included repeated reporting of alcohol consumption throughout the night and the provision of tailored messages which could correspond to surveys they filled in during the night. Participants were then engaged in a facilitated discussion relating to intervention design.

The workshops had four stages: (1) a focus-group style discussion on drinking habits and motivations, (2) discussion of the design features of the intervention, (3) analysis of existing media campaigns related to alcohol, and (4) a development component in which participants generated their own message content and gave further design feedback.

First, participants were asked to describe a “typical” night out, including usual drinking patterns and their as well as their peers’ event-level behavior. They also discussed their own motivations, if any, for reducing alcohol consumption within drinking events as well as more broadly, including what intervention features they felt would motivate them, and separately, what they felt would motivate their peers and young people in general. The groups also identified the types of health promotion content they found acceptable and relevant. Message style, language, framing, and topics were discussed. Participants reflected on tailoring required for different genders and ages, as well as event-specific contexts (eg, messages which might apply when drinking at a bar but not at private venues and messages relevant to stages of the night).

Second, participants were asked questions relating to the design of the intervention including ideal platforms, frequency of data collection and message delivery, questionnaire items, and feasibility. The results of this specific component of the study are reported elsewhere [52].

Third, participants were engaged in a media content analysis of over 20 diverse alcohol-related campaigns from Australia and elsewhere. These examples were taken from previous studies and public campaigns that included short alcohol messages. We selected examples which represented different message communication approaches, with a combination of text and image-based formats. Participants were shown examples one-by-one on a projector and asked to discuss reactions, comprehension, relevance, persuasion, and attractiveness. We asked which, if any, examples would be useful and appropriate and how to modify those that they thought were potentially useful. Participants discussed how best to translate messages for mobile phone and in-moment delivery, including format, length, topic appropriateness, and language.

Finally, participants were divided into groups of 2 to 4 people and asked to develop their own content for messages to be sent at different stages of their typical night out, based on topics that they felt would be relevant to them. Participants were given activity sheets developed by the researchers on which to list content and ideas.

### Analysis

The sessions were recorded digitally and the recordings transcribed verbatim. Four digital recorders were used in each session, so that any conversations which occurred during small group work could be captured. All transcripts from workshops, as well as notes written by participants, were analyzed thematically using NVivo 10 Software (QSR International Pty Ltd.) [53]. An inductive approach was used, whereby a coding framework was developed using the raw qualitative data that was iteratively refined during the analysis process. Two researchers separately coded an initial sample of transcripts and checked consistency. Only minor discrepancies emerged and were resolved by discussion; the remaining transcripts were coded by a single researcher. Data from all workshop components were combined for analysis and coded as relevant to the various codes and themes.

## Results

In total, 42 people attended the development workshops—21 women and 21 men aged 18-25 years. Findings were divided into two categories: (1) the style of messages preferred for delivery during a drinking event, and (2) the topics that participants considered were appropriate for this type of intervention.

### Style of Messages

Participants were asked about the preferred style and tone of the intervention. Without prompting or leading, almost all agreed that in order for content to be acceptable, nonjudgmental and non-authoritative language was essential. Participants described getting into a different state of mind when they commenced a drinking episode. This seemed to involve a concentrated focus on having fun and blocking out any incongruent negative influences on their night. This is an important idea with respect to any intervention occurring during a social event. Participants also advocated for some messages to be framed as questions, so as to allow them to reflect on their own behavior without being told by someone else why they should change. One participant felt that this would be more effective if applied to a message sent the day following the event:

At the time, if you are drinking, if you are doing things, you are going to be like, “I’m so awesome, I’m doing this thing,” and then the next day you go, “that was not good. Everyone is going to be remembering that. I’m going to remember that. That was awful.” I think it would be more the next day because if you were regretting it at the time, you probably wouldn’t maybe do it.

Participants requested that some positive reinforcement be provided during the intervention to create a more positive interaction. One participant communicated the desire for an intervention to “tell me what I’m doing right.” This sentiment was echoed across the workshops, with participants frequently mentioning the need for more encouraging messaging.

Fear-based campaigns or messages were described as likely to be ineffective as participants of both genders felt easily able to dismiss the seriousness or relevance of harms while in a social context. The following statements exemplify a common attitude among the participants:

I don’t know anyone who got drinking-related cancer...

...people pass out all the time...

If you get alcohol poisoning then they just pump your stomach and you’re fine the next day.

A clear preference for short, actionable harm-reduction focused messages was observed, including suggestions for reminders around drinking water, organizing a ride home, checking on friends, eating enough, and reminders of plans the next day. One participant articulated these points as follows:

If you tell me I’ll get sick when I’m old...What do I care? I can’t change that anyway. It’s the short-term stuff I can do something about.

Both male and female participants were excited about the potential for topics to be tailored to individual preference, as they felt many of the recent public alcohol-related campaigns were too generic and often irrelevant to them and their peers. Participants were keen for the messages to provide genuinely tailored feedback based on their reported preferences and behaviors, such as reminders based on their reported plans, tracking of cumulative drinking and spending, and the reflection of their own personally reported motivations.

### Message Topics

Our participants reported that messages based on drinking norms messages were largely irrelevant to them; they felt they had a strong grasp on the norms of their social circles and were not concerned about what was normal to other young people. In each group, regardless of gender, the messages provided to participants from the norms-based example campaign were contested by at least one participant who did not believe the statistics presented were accurate. This was seen to compound the preexisting idea held by many participants that researchers and practitioners were “out of touch” with young people’s needs. A number of male participants touched on the idea that norms-based campaigns could have an effect opposite to that intended because of the “proud” culture around excessive drinking in Australian males:

Nah, and on the contrary, I reckon you would be like, “YOLO! (You Only Live Once). We do this.” I reckon...Yeah, that doesn’t resonate with me at all.

Indeed, most participants from our workshops claimed that they already knew about the harms related to alcohol consumption, but they did not seem serious or relevant in a social context. This did not seem to differ by gender. Unsurprisingly, long-term harms were described as especially unmotivating, despite many of the participants not knowing about, for example, the cancer-related harms of drinking. “Wouldn’t even read that—buzz killer, etc.”; “I’d just ignore it. Everything gives you cancer, may as well just have fun.” Instead, participants felt they would be most motivated by avoiding the consequences that they themselves had previously experienced—such as hangovers, losing possessions, vomiting, and memory loss—and therefore messages to aid the avoidance of these proximal harms were seen as useful.

Social burden was also seen as relevant: young people didn’t want to let down their peers or “ruin the night.” This sentiment was the same across genders, although described in a slightly different language. Participants in the male-only focus group discussed extensively the stereotype of the “shit mate,” or “maggot” who would “cut loose” at the expense of others. They recounted regretful episodes where they or their friends had drunkenly started fights, passed out and been too heavy to move, had been ejected from nightclubs, or had vomited in cars. Females across both the single-gender group and mixed groups more commonly described wanting to avoid being “that messy girl” who cried, whose makeup was smudged, and who needed greater protection and supervision. Safety and protection from others was a key concern for females and for female friends, whereas protection of males was more likely to relate to stopping them from hurting themselves by engaging in a risky behavior or violence.

You all have that one mate that just gets agro and you’re like oh god, where’s he gone, what’s he doing now—have to pull him away non-stop.

One female participant described that sending messages relating to checking on friends could serve the dual purpose of encouraging them to sober up in order to be able to protect their friends, while simultaneously reminding them not to be that drunk person themselves:

When you drink, or when I do, and your friend is—[pause]. You all of a sudden become like this protective person that wants to help. I think a lot of people do, if you love your friends or whatever [laughter]. The protecting your friends part is important. Even, “Check on your friends, how are they going?” because it could make you look at them and be like, wait, okay, this person is acting weird. Maybe I should tone down so I can help. But also you don’t want to be that weird friend either.

Spending was seen as a key motivator, as young people reported that they often experienced financial hardship and felt inexperienced at sticking to a budget. Some participants suggested the use of diet and exercise-based messages; across all focus groups, there was a clear gender difference with females more supportive of diet-based messages and males more supportive of sporting-related messages. A suggested form of feedback was comparing calories consumed as alcohol with those as junk food: “How many cheeseburgers am I drinking?” A male participant advocating for an exercise message reported: “I have footy (football) every Saturday, so if you reminded me about that...“However not all participants were interested in diet and exercise messages, and a few females expressed concern for the unintended consequences of this message type. “If you sent me that then I probably would just skip dinner instead.” Another female participant worried that it may even encourage existing disordered behavior:

The only thing with that is can it make people who are really insecure, anxious. It makes you not want to eat...You could get that random one person that gets it and she is like, "Oh no, I’m going to go and throw up."

## Discussion

### Principal Findings

Without any prompting, young people advocated for a style very similar to motivational interviewing in approach involving four basic strategies: open-ended questions, affirmations, reflective statements and summary statements [11]. These characteristics were all raised by study participants as preferable, as were nonjudgmental framing and emphasis on personally derived motivations. These findings are in line with other relevant research [37,42,51,54], although previous studies not always described in the context of brief intervention or motivational interviewing theory [37,42]. Preferences for intervention message content do not appear to have been explored in the context of delivery during a drinking event. The findings from this development study suggest that brief intervention and motivational interviewing principles may be important considering this social context.

Our participants dismissed some previous alcohol intervention strategies and campaigns as unappealing due to their focus on health. This finding is aligned with previous studies such as that of de Visser et al who found that their young participants were generally unconcerned by health consequences and more motivated by social factors [51]. Contrary to this, Riordan et al found than both men and women preferred messages focusing on long-term health over other types of alcohol consequences [50], although social factors also rated highly. Our participants’ more firm rejection of consequence-based or negative messages may reflect the social context of our intervention timing. Alternatively, it could reflect a push back from the emphasis on fear appeal which has been predominantly preferred in Australian alcohol campaigns. Although there is some evidence supporting the use of fear-based messages in specific contexts and topics, they are usually coupled with strategies that affect policy or environments [55-57]. Graphic drink-driving mass-media campaigns in Australia are often cited as successful fear-based messaging, but in reality these advertisements represent only one part of a multipronged effort that also includes policy and enforcement [56,57]. Given that there is no equivalent or relevant “enforcement” strategy with which to pair our intervention messages, fear-based messages are unlikely to be effective. Thomas et al similarly found in their formative work to develop an alcohol intervention that participants warned against the use of “scare tactics,” expressing that these types of messages may induce anxiety or guilt and cause them to disengage [37]. Hospital et al described a strong consensus from participants that content for alcohol interventions should have an overly positive tone [54].

Findings relating to motivations for drinking less, such as burdening friends, have also been discussed in previous studies [50,51]. De Visser et al [51] and Riordan et al [50] both similarly found that young people were strongly motivated by wanting to avoid ruining their friends’ night. Our study adds to the idea of using protection of friends to motivate young people to drink less. One previous study found that a message encouraging young people to look out for their friends was among the highest-rated of their expert-authored messages [47]. One previous study also found the same gendered differences in relation to safety and protection, with concerns for protecting females from others, and males from themselves [51]. These socially related motivations could make for a key target for in-event intervention delivery due to their broad relevance.

Diet and exercise-based messages have been used in some recent public alcohol-related campaigns, but in light of this study and other relevant findings, there is a need for caution to ensure that unintended harm is not caused. Knight [58] recently reported “drunkorexia,” meaning skipping meals to allow consumption of more calories as alcohol, in young Australian women [58,59]. Our participants reporting this type of behavior, along with the high prevalence and underdiagnosis of eating disorders in Australian young people [60], was a central reason for minimizing use of diet and exercise messages in our intervention. Two exceptions were reminders early in the night to encourage eating a meal before drinking and a reminder during the night if a participant reported that they had sport or exercise planned for the following morning.

Reactions to normative-based messages were surprising, considering that these are an increasingly common feature of alcohol brief interventions [31-33,60-62]. Whereas a great deal of research shows that peer and social norms are important determinants of drinking behavior, there is less evidence for the effectiveness of social norms information being used in interventions to reduce drinking. A recent Cochrane review found only small effects on drinking in interventions which used this approach; however, authors noted that these studies were of low or moderate quality [62]. In our study, the use of norms-based feedback was seen as irrelevant when participants had contradictory real-life norms and experiences. This perception of irrelevance may be heightened in an in-moment intervention, when “contradictory behavior” is occurring during intervention delivery. It is possible that normative-based feedback needs to be context-specific or more highly tailored in order to feel relevant to the recipient and be effective. Participants also introduced the concerning idea that norms of drinking to excess were a source of pride and may be therefore ineffective even if relatable. The application of this message type for in-moment interventions requires further investigation.

Preference for short and actionable messages is well suited to the mobile platform and the in-moment delivery of messages. However, the style and tone of these short messages is important for acceptability [37,54]. Thomas et al also found that young people preferred alcohol-related text messages that were succinct, clear, and encouraging [37].

### Limitations

This study used a small, nonprobability sample, but generalizability is not a focus of this qualitative research. Deep and rich insights are more important in this context, given how little is known about how to intervene during risky drinking events. Considering how new and emerging this area is, we could have chosen to employ in-depth interview methods instead, to allow deeper probing with each participant. We chose to use focus groups not only because of resource constraints but also due to the benefit of idea generation and examination of consensus which can occur in groups. At the completion of data collection, some new ideas were still emerging and some researchers may have chosen to continue collecting data. Our team decided that our main research questions had been answered with enough consistency across the four groups that we could be confident in our decision to close data collection. Acceptability might not equal behavior change. The efficacy of our intervention and the message content developed in these workshops in reducing alcohol consumption has not yet been tested; this will be the subject of future research.

### Conclusions

Recent research has attempted to harness technology to deliver brief interventions via mobile phone platforms, including for alcohol harm reduction. Although this innovation offers new opportunities, there is a need for improved content development processes (such as use of theory and participatory research), as well as transparency in reporting these processes.

Findings from this study offer insights into young adults’ drinking events, as well as practical advice for designing alcohol-related brief interventions. During our formative development process, we demonstrated a neat correspondence between young people’s preferences for alcohol-reduction interventions and the theoretical principles of both brief interventions and motivational interviewing, including acceptable topics and message style. It is recommended that creators of future mobile brief interventions look beyond the basic “feedback” component of brief interventions and consider integrating more of the FRAMES model components in order to maximize both the acceptability and theory-base of their interventions. Delivery of interventions during risky events such as drinking alcohol also offers new opportunities, but careful consideration of the context is required when designing message content to maximize its effectiveness. In order to advance the evidence base for alcohol brief interventions delivered by SMS, further work is needed to test differences in brief intervention types, including varying approaches to messaging within interventions.

## Abbreviations

FRAMES model: Feedback, Responsibility, Advice, Menu of options, Empathy, and Self-efficacy
